# CD73 represses pro-inflammatory responses in human endothelial cells

**DOI:** 10.1186/1476-9255-7-10

**Published:** 2010-02-05

**Authors:** Jana KG Grünewald, Anne J Ridley

**Affiliations:** 1 King's College London, Randall Division of Cell and Molecular Biophysics, New Hunt's House, Guy's Campus, London SE1 1UL, UK

## Abstract

**Background:**

CD73 is a 5'-ectonucleotidase that produces extracellular adenosine, which then acts on G protein-coupled purigenic receptors to induce cellular responses. CD73 has been reported to regulate expression of pro-inflammatory molecules in mouse endothelium. Our aim is to determine the function of CD73 in human endothelial cells.

**Methods:**

We used RNAi to deplete CD73 levels in human umbilical cord endothelial cells (HUVECs).

**Results:**

CD73 depletion resulted in a strong reduction in adenosine production, indicating that CD73 is the major source of extracellular adenosine in HUVECs. We find that CD73 depletion induces a similar response to pro-inflammatory stimuli such as the cytokine TNF-α. In CD73-depleted cells, surface levels of the leukocyte adhesion molecules ICAM-1, VCAM-1 and E-selectin increase. This correlates with increased translocation of the transcription factor NF-kB to the nucleus, which is known to regulate ICAM-1, VCAM-1 and E-selectin expression in response to TNF-α. Adhesion of monocytic cells to endothelial cells is enhanced. In addition, CD73-depleted cells become elongated, have higher levels of stress fibres and increased endothelial permeability, resembling known responses to TNF-α.

**Conclusions:**

These results indicate that CD73 normally suppresses pro-inflammatory responses in human endothelial cells.

## Background

CD73 is a 5'-ectonucleotidase that uses extracellular AMP to produce adenosine, and is a GPI-anchored protein that is expressed abundantly on endothelial cells and on a subset of leukocytes [1,2]. CD73^-/- ^mice are viable but have multiple cardiovascular phenotypes [3], including cardioprotection during myocardial ischemia [4], vasoprotection [3,5], increased neointimal plaque formation and increased monocyte adhesion due to upregulation of VCAM-1 on the endothelium [6]. In the cremaster model of ischaemia-reperfusion, leukocyte attachment to the endothelium is significantly increased in CD73^-/- ^mice [3]. Additionally, CD73^-/- ^mice have increased vascular leakage in response to hypoxia [5], lipopolysaccharide (LPS) [7] and cardiac transplantation [8]. Whether these phenotypes are a consequence of reduced adenosine production by endothelial or other cell types is not known, although inhibition of CD73 enzymatic function induces a similar accumulation of neutrophils in lungs following LPS treatment to lack of CD73 [7].

Adenosine generally has anti-inflammatory properties and exerts its effects via G-protein-coupled P1 purinergic receptors [2], although in some cell types purinergic receptors play a pro-inflammatory role [9]. A_2A _and A_2B _purinergic receptors activate adenylate cyclase, thereby increasing intracellular cAMP levels, while A_1 _and A_3 _receptors inhibit cAMP production [10]. In endothelial cells, stimulation of A_2B _receptors increases endothelial barrier function by decreasing actomyosin contractility and strengthening the intercellular junctions [11,12], and A_2B_-null mice have increased vascular permeability in response to hypoxia and increased pulmonary leakage after lung injury [13,14]. Adenosine has also been shown to inhibit neutrophil adhesion to the endothelium and transendothelial migration via neutrophil A_2 _receptors [15,16], and an inhibitor of CD73-mediated adenosine production was found to enhance migration of lymphocytes across brain microvascular endothelial cells [17]. CD73 is therefore proposed to provide an anti-inflammatory signal via adenosine production, leading to increased endothelial barrier function and decreased leukocyte binding.

In addition to increasing endothelial barrier function, adenosine inhibits NF-κB-mediated upregulation of leukocyte adhesion molecules on endothelial cells including P-selectin, E-selectin and VCAM-1 [18-21]. The regulation of ICAM-1 by adenosine is unclear; while Bouma et al. did not see an adenosine-mediated decrease in ICAM-1 levels [22], others have demonstrated inhibition of ICAM-1 expression in response to adenosine analogues or A_2A _receptor agonists [18,21].

Although adenosine has multiple affects in protecting human endothelial cells from pro-inflammatory stimuli and CD73 produces adenosine, whether endogenous CD73 contributes to endothelial cell function in the absence of pro-inflammatory stimuli is not clear. In order to investigate how CD73 affects the properties of human endothelial cells, we have used RNAi to reduce CD73 expression. We show that CD73 depletion induces a phenotype similar to that of the pro-inflammatory cytokine TNF-α, including upregulation of leukocyte adhesion molecules, changes to cell shape and the actin cytoskeleton, and increased endothelial permeability.

## Methods

### Reagents

Human fibronectin, adenosine 5'-monophosphate, TRITC-phalloidin and FITC-dextran (Mr 42 000) were obtained from Sigma-Aldrich; Oligofectamine reagent, AlexaFluor594-labelled goat anti-rabbit and AlexaFluor488-labelled goat anti-mouse antibodies were obtained from Invitrogen; mouse anti-CD73 antibody (4G4) was a gift from Sirpa Jalkanen (Turku, Finland); mouse anti-ICAM-1 antibody (BBIG-I1) was from R&D Systems; mouse anti-VCAM-1 antibody (51-10C9) and mouse anti-β-catenin (AC15) were from BD Pharmingen; mouse anti-E-selectin (CTB202) and rabbit anti-NF-κB (p65) antibody (C-20) were from Santa Cruz Biotechnology; [2-^3^H] adenosine 5'-monophosphate was obtained from GE Healthcare.

### Cell Culture

Pooled human umbilical vein endothelial cells (HUVECs) were obtained from Lonza and cultured in flasks pre-coated with 10 μg/ml human fibronectin in EBM-2 medium with growth factors (Lonza) in an atmosphere of 5% CO_2 _and 95% air. The human monocytic cell line THP-1 (ATCC) was cultured in RPMI-1640 medium (Invitrogen) supplemented with 2 mM L-glutamine, 10% heat-inactivated fetal calf serum (FCS), penicillin (100 U/ml) and streptomycin (100 μg/ml) in an atmosphere of 5% CO_2 _and 95% air.

### siRNA Transfection

HUVECs were plated on 6-well dishes at 1.5 × 10^5 ^cells per well, 24 h prior to transfection. siRNAs (1.25 μl of 20 μM stock) were premixed with 4 μl of Oligofectamine reagent (Invitrogen). The three siRNAs oligonucleotides si1, si2 and si3 targeting human NT5E (CD73) were siGENOME duplexes D-008217-01 (GAACCUGGCUGCUGUAUUGUU), D-008217-02 (GGAAGUCACUGCCAUGGAAUU) and D-008217-04 (GGACUUUAUUUGCCAUAUAUU) (Dharmacon). The non-targeting control siRNA (siC) was ON-TARGETplus D-001810-01 (UGGUUUACAUGUCGACUAA). Cells were transfected for 4 h at 37°C in 1 ml EBM-2 medium with growth supplements but no antibiotics or FCS. EBM-2 medium (0.5 ml) with growth factors and 6% FCS was then added to each well and cells were incubated over night. Cells were trypsinized 48 h after transfection and plated on fibronectin-coated 6-well plates (4 × 10^5 ^cells per well; flow cytometry or phase-contrast images), 24-well plates (2 × 10^5 ^cells per well; thin layer chromatography), coverslips (2 × 10^5 ^cells per coverslip; immunofluorescence), black 96-well plates with glass bottom (5 × 10^4 ^cells per well; adhesion assay) or Transwells (2 × 10^5 ^cells per Transwell; permeability assay). Where indicated, cells were stimulated with 10 ng/ml TNF-α for 15 h. Cells were analyzed 72 h after transfection.

### Flow Cytometry

Flow cytometry (FC) was used to detect levels of cell surface receptors in HUVECs. Cells were detached with trypsin/EDTA and washed once with FC flow buffer (0.2% BSA, 0.1% N_3_Na in PBS). Cells were then sequentially incubated with 2% BSA in FC buffer (30 min, 4°C), primary antibody (30 min, 4°C) and AlexaFluor488-conjugated goat anti-mouse antibody (20 min, 4°C). To remove the antibodies, cells were washed twice with FC buffer. Samples were measured using a BD FACSCalibur flow cytometer (Becton Dickinson) at 488 nm excitation wavelength and using a 530 nm emission bandpass filter.

### CD73 Activity Assay

HUVECs were washed once before adding EGM-2, containing 180 μM [2-^3^H] adenosine 5'-monophosphate (specific activity per well: 37 μBq) and 200 μM unlabelled adenosine 5'-monophosphate (10 min, 37°C). Aliquots of the medium were applied to silica gel 60 ADAMANT™ thin layer chromatography (TLC) plates (Sigma-Aldrich) and were separated using isobutyl alcohol:isoamyl alcohol:2-ethoxyethanol:ammonia:H_2_O (ratio 9:6:18:9:15) as a solvent. The TLC plates were developed by exposing to tritium-sensitive film (Kodak BioMax MS film) together with a BioMax TranscreenLE intensifying screen (Kodak). TLC spots were quantified by densitometry and relative CD73 activity was calculated as ^3^H-adenosine/^3^H-AMP.

### Immunofluorescence and Phase-contrast Microscopy

HUVECs were washed once with PBS and fixed with 4% paraformaldehyde in PBS (20 min, room temperature) and for NF-κB localisation additionally with 100% ice-cold acetone (5 min, -20°C). After fixation cells were permeabilised with 0.1% Triton X-100 in PBS (5 min, 4°C) and blocked with 2% BSA in PBS (30 min, 22°C). Coverslips were then sequentially incubated with antibodies against NF-κB (p65) and β-catenin, AlexaFluor488 goat anti-mouse and AlexaFluor594 goat anti-rabbit antibodies and/or with TRITC-phalloidin to visualise F-actin (45 min, 22°C). Coverslips were mounted onto slides using fluorescent mounting medium, and visualised using a LSM 510 laser scanning confocal microscope (Zeiss). Phase-contrast images of siRNA-treated HUVECs in 6-well dishes were generated on a Nikon Eclipse TE2000-E microscope with a Hamamatsu Orca-ER digital camera using Metamorph software.

### Cell Adhesion Assay

THP-1 cells were stained with CellTracker Green CMFDA (1 μM, 30 min, 37°C), washed once with PBS and 5 × 10^6 ^THP-1 cells were added for 15 min to black 96-well dishes with clear bottom (Corning) containing siRNA-treated HUVECs. The wells were washed twice with PBS and the remaining fluorescence measured in a Fusion α-FP plate reader (Perkin Elmer) at 485 nm excitation wavelength and using a 525/35 nm emission bandpass filter.

### Permeability Assay

siRNA-treated HUVECs were cultured to confluency on Transwell filters (Corning; 12 mm diameter, 0.4 μm pore size), cells were washed once with medium and 100 μg/ml FITC-dextran was applied to the upper chamber. Samples of the medium from the lower chamber were subsequently removed after 80 min and measured in black clear-bottom 96-well plates using a Fusion α-FP plate reader (Perkin Elmer) at 485 nm excitation wavelength and using a 525/35 nm emission bandpass filter.

### Statistical Analysis

In order to determine statistical significance, Student's t-test with Bonferroni post-test was carried out using GraphPad Prism software http://www.graphpad.com.

## Results

### CD73 is the main source of adenosine production by HUVECs

**Figure 1:**
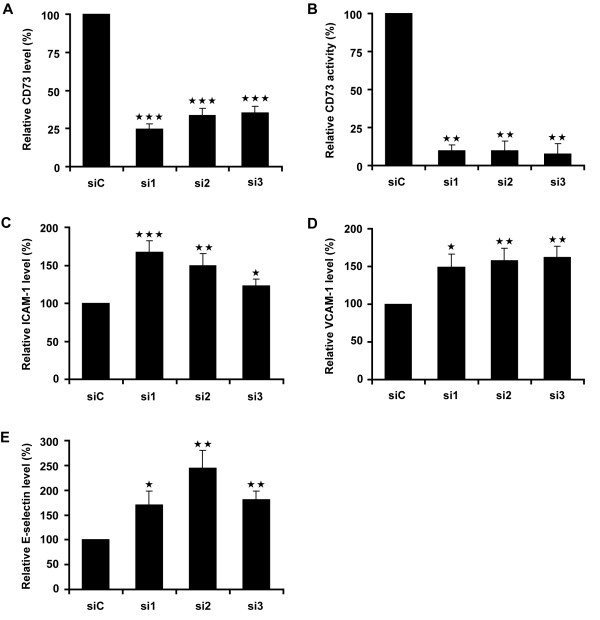
**CD73 regulates ICAM-1, VCAM-1 and E-selectin expression**. HUVECs were transfected with CD73 siRNAs or control oligonucleotide (siC). **A**, Cell surface expression levels of CD73. **B**, CD73 activity. **C-E**, ICAM-1, VCAM-1 and E-selectin, shown as mean fluorescence of the population. Results were normalised to siC. ***p < 0.001, **p < 0.01, *p < 0.05 determined by Student's t-test and Bonferroni post-test, compared to siC.

To investigate the role of CD73 in human endothelial cells, HUVECs were transfected with three different siRNAs to CD73 (si1, si2 and si3), all of which reduced surface levels of CD73 by at least 70%, whereas a control non-targeting siRNA (siControl; siC) did not affect CD73 levels (Figure 1A). Adenosine is the product of CD73 enzymatic activity. It was constitutively produced by HUVECs, and this was markedly reduced in CD73 knockdown cells (Figure 1B), indicating that CD73 is the major source of extracellular adenosine in these cells.

### CD73 regulates adhesion molecule expression in endothelial cells

Pro-inflammatory cytokines up-regulate the expression of the leukocyte adhesion molecules ICAM-1, V-CAM-1 and E-selectin in endothelial cells [19]. To investigate whether CD73 regulates cell surface levels of these adhesion molecules, we tested the effects of CD73 depletion. Unstimulated HUVECs expressed low levels of ICAM-1 on the cell surface, whereas VCAM-1 and E-selectin levels were not above background (data not shown). CD73 depletion induced an increase in ICAM-1, VCAM-1 and E-selectin levels, whereas siControl had no effect (Figure 1C-E). Taken together, these results are consistent with a role of constitutive adenosine production by CD73 in suppressing expression of leukocyte adhesion molecules in endothelial cells.

**Figure 2:**
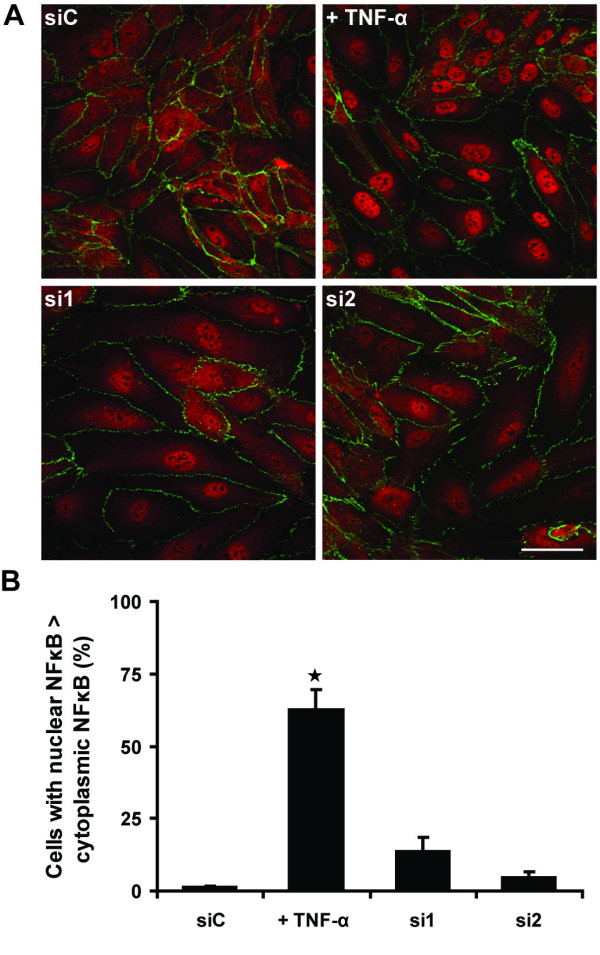
**CD73 depletion increases nuclear localisation of NF-κB**. HUVECs were transfected with CD73 siRNAs or control siC. **A**, Immunolocalization of NF-κB (p65) and β-catenin. Bar = 50 μm. **B**, Quantification of NF-κB localization; at least 100 cells were counted in each of three independent experiments. * p < 0.05 determined by Student's t-test and Bonferroni post-test, compared to siC.

TNF-α induces ICAM-1, VCAM-1 and E-selectin expression in part through activation of the transcription factor NF-κB [19]. NF-κB activity was reported to be increased in endothelial cells derived from CD73^-/- ^mice, and thus could contribute to upregulation of VCAM-1 levels [6]. To test if NF-κB activity was increased in HUVECs depleted of CD73, cells were stained with antibodies to NF-κB. NF-κB translocates to the nucleus when it is activated [23], and TNF-α, which is well known to stimulate NF-κB activity, stimulated NF-κB nuclear translocation in over 60% of HUVECs (Figure 2). CD73 depletion also increased the proportion of cells with nuclear NF-κB staining (Figure 2). These results suggest that CD73 knockdown induces a pro-inflammatory phenotype in HUVECs, which could be mediated in part by NF-κB activation.

### CD73 depletion induces morphological changes in HUVECs

**Figure 3:**
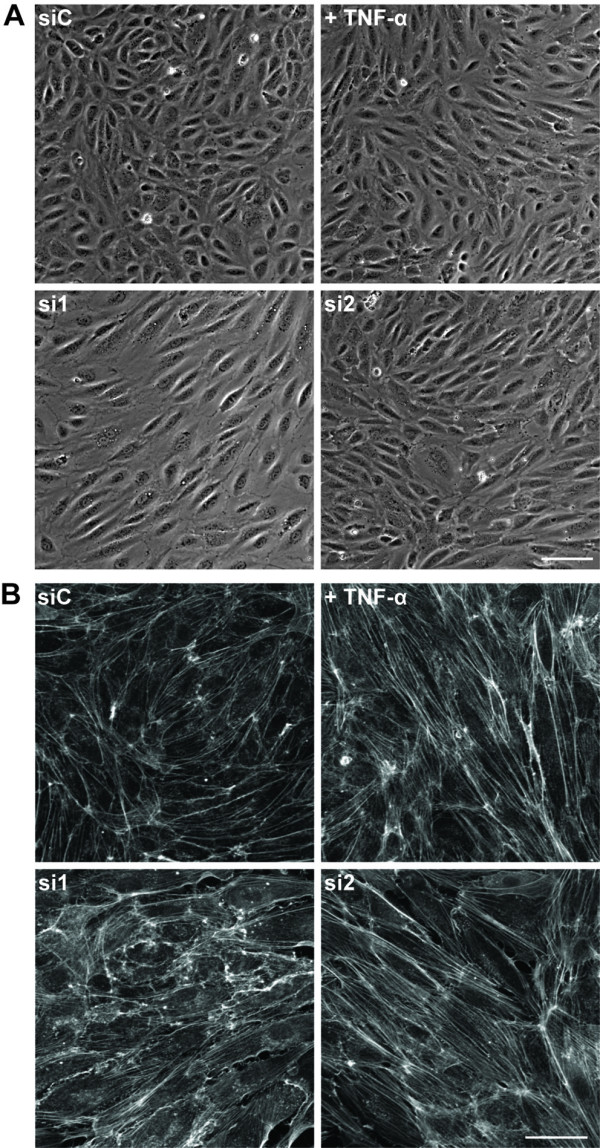
**CD73 regulates endothelial morphology**. HUVECs were transfected with CD73 siRNAs or control oligonucleotide (siC), and stimulated with or without TNF-α. Representative phase-contrast images (A) and confocal images of actin filaments (B) of at least five independent experiments are shown. Bars = 50 μm.

Since CD73 knockdown induced upregulation of adhesion molecules similar to TNF-α, we tested whether CD73 affected endothelial morphology. We have previously shown that TNF-α induces cell elongation and actin stress fibre formation in HUVECs [24]. CD73 knockdown induced an elongated morphology similar to morphological changes occurring after TNF-α treatment (Figure 3). CD73 depletion also increased stress fibres, although to a lesser extent than 10 ng/ml TNF-α (Figure 3). These results further strengthen the hypothesis that CD73 depletion induces a pro-inflammatory phenotype.

### CD73 regulates leukocyte adhesion

**Figure 4:**
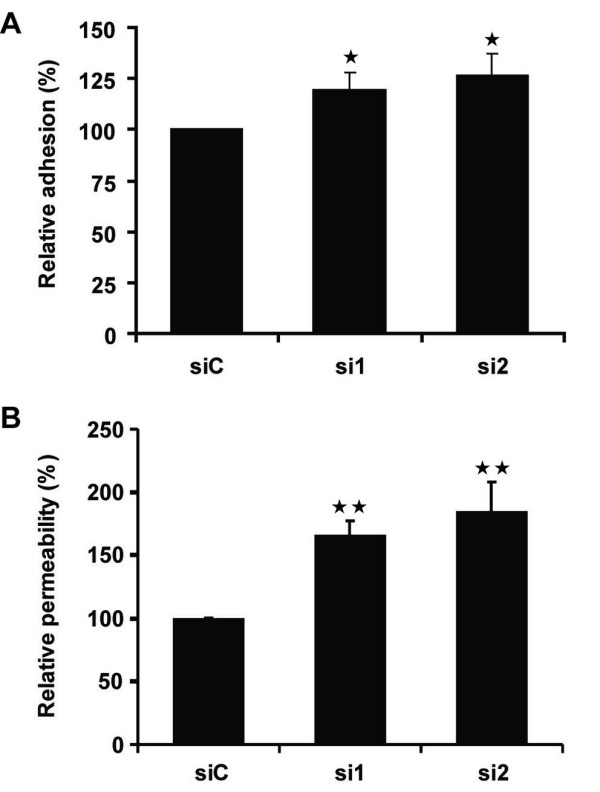
**CD73 depletion increases monocyte adhesion to endothelial cells and endothelial permeability**. HUVECs were transfected with CD73 siRNAs or control siC. A, Adhesion of THP-1 cells to HUVECs was measured after 15 min. B, Monolayer permeability was determined on Transwell filters. Results were normalised to the respective control (siC). **p < 0.01, *p < 0.05, determined by Student's t-test and Bonferroni post-test, as compared to siC.

The increase in adhesion molecule expression in CD73-depleted endothelial cells suggests that leukocyte adhesion could be affected. To study this we incubated THP-1 monocytic leukaemia cells with HUVECs. Adhesion of THP-1 cells to HUVECs was significantly increased by CD73 knockdown (Figure 4A). In contrast, CD73 depletion did not affect THP-1 adhesion to TNF-α-treated HUVECs, reflecting the 4 to 6 fold increase in the levels of ICAM-1, VCAM-1 and E-selectin expression induced by TNF-α alone (data not shown).

### Endothelial permeability is increased in CD73-depleted cells

TNF-α is known to increase endothelial permeability in HUVECs [24,25], whereas adenosine, the product of CD73 enzymatic activity, has been shown to reduce permeability [11,12,26]. The decrease in extracellular adenosine production due to CD73 knockdown (Figure 1C) would therefore be predicted to lead to an increase in permeability. In agreement with this, the permeability of HUVEC monolayers was higher following CD73 depletion than in control cells (Figure 4B). The 1.5 to 2-fold-increase in permeability following CD73 knockdown was in the same range to that induced by 10 ng/ml TNF-α (2 to 2.5 fold; data not shown and [24])

## Discussion

The endothelium of CD73^-/- ^mice has been shown to have increased VCAM-1 levels, but the effect of CD73 depletion on human endothelial cells has not been described. We show here that CD73 normally functions to suppress multiple different aspects of a pro-inflammatory phenotype of endothelial cells, including expression of ICAM-1, VCAM-1 and E-selectin, translocation of the transcription factor NF-κB to the nucleus, endothelial cell morphology, actin cytoskeletal organisation and permeability. CD73-depleted cells exhibited a similar phenotype to treatment with TNF-α.

Consistent with the lower levels of leukocyte adhesion molecules and leukocyte adhesion we observe in CD73-depleted endothelial cells, leukocyte infiltration in inflammatory situations is reduced in CD73^-/- ^mice [7,27,28]. Endothelial CD73 is important for these responses [28], although lymphocyte CD73 also contributes to reducing cardiac graft rejection [8]. In lymphocytes it has been suggested that CD73 has non-enzymatic functions in modulating the clustering of the integrin LFA-1 or in inhibiting apoptosis, but so far no such role of CD73 has been described in endothelial cells [1,29]. However, an A_2B _adenosine receptor agonist rescues the defect in lymphocyte recruitment to lymph nodes in CD73^-/- ^mice [28], indicating that in this case the phenotype is probably due to decreased levels of adenosine.

It is likely that the signalling pathway whereby CD73 and adenosine suppress leukocyte adhesion molecule expression differs from that regulating morphology and endothelial permeability. The regulation of endothelial permeability and stress fibre levels by adenosine is attributed to an increase in cAMP, which in turn induces both inhibition of RhoA, and hence decreases actomyosin contractility and stress fibre formation, and activation of Rap1, thereby strengthening adherens junction integrity [30]. Although the mechanistic basis for adenosine-mediated inhibition of leukocyte adhesion molecule expression is less clear, it is possible that it also involves cAMP production, since increased cAMP inhibits TNF-α-and thrombin-induced transcription of NFκB-regulated genes, including ICAM-1 and VCAM-1 [31,32], an effect that could be mediated through cAMP-induced repression of p38 MAPK activity [31].

It is not clear whether the pro-inflammatory phenotypic changes we observe in response to CD73 depletion represent the constitutive activity of an intrinsic signalling pathway in endothelial cells that is suppressed by CD73 and adenosine or are mediated by an external stimulus. It is possible that HUVECs themselves produce some TNF-α or other pro-inflammatory cytokines, although TNF-α production by endothelial cells is normally only induced by inflammatory stimuli such as LPS or interleukin 1β [33,34]. In the future it would be interesting to determine whether the anti-inflammatory effects of CD73 are mediated by alterations in the constitutive activity of GTPases such as RhoA or Rap1. It will also be important to investigate whether the effects of reduced CD73 expression we report with human endothelial cells in vitro correlate with in vivo observations on human endothelium.

## Conclusions

CD73 depletion in HUVECs induces a pro-inflammatory phenotype similar to low levels of TNF-α, including increased expression of leukocyte adhesion molecules and changes in endothelial morphology. Since we found that HUVECs normally produce extracellular adenosine and that this is predominantly due to CD73, it is likely that reduced levels of adenosine are responsible for the phenotypes we observe upon CD73 knockdown.

## Competing interests

The authors declare that they have no competing interests.

## Authors' contributions

JKGG and AJR designed the study. JG carried out all experimental work and prepared the figures. JKGG and AJR wrote the manuscript. Both authors have read and approved the final manuscript.
